# An automatic deep reinforcement learning bolus calculator for automated insulin delivery systems

**DOI:** 10.1038/s41598-024-62912-4

**Published:** 2024-07-02

**Authors:** Sayyar Ahmad, Aleix Beneyto, Taiyu Zhu, Ivan Contreras, Pantelis Georgiou, Josep Vehi

**Affiliations:** 1 Modeling and Intelligent Control Engineering Laboratory, Institute of Informatics and Applications, University of Girona, 17003 Girona, Spain; 2 Centre for Bio-Inspired Technology, Department of Electrical and Electronic Engineering, Imperial College London, London, UK; 3 Centro de Investigación Biomédica en Red de Diabetes y Enfermedades Metabólicas Asociadas (CIBERDEM), 28001 Madrid, Spain

**Keywords:** Automatic insulin delivery, Artificial pancreas, Unannounced meals, Deep reinforcement learning, Biomedical engineering, Computer science

## Abstract

In hybrid automatic insulin delivery (HAID) systems, meal disturbance is compensated by feedforward control, which requires the announcement of the meal by the patient with type 1 diabetes (DM1) to achieve the desired glycemic control performance. The calculation of insulin bolus in the HAID system is based on the amount of carbohydrates (CHO) in the meal and patient-specific parameters, i.e. carbohydrate-to-insulin ratio (CR) and insulin sensitivity-related correction factor (CF). The estimation of CHO in a meal is prone to errors and is burdensome for patients. This study proposes a fully automatic insulin delivery (FAID) system that eliminates patient intervention by compensating for unannounced meals. This study exploits the deep reinforcement learning (DRL) algorithm to calculate insulin bolus for unannounced meals without utilizing the information on CHO content. The DRL bolus calculator is integrated with a closed-loop controller and a meal detector (both previously developed by our group) to implement the FAID system. An adult cohort of 68 virtual patients based on the modified UVa/Padova simulator was used for in-silico trials. The percentage of the overall duration spent in the target range of 70–180 mg/dL was $71.2\%$ and $76.2\%$, $<70$ mg/dL was $0.9\%$ and $0.1\%$, and $>180$ mg/dL was $26.7\%$ and $21.1\%$, respectively, for the FAID system and HAID system utilizing a standard bolus calculator (SBC) including CHO misestimation. The proposed algorithm can be exploited to realize FAID systems in the future.

## Introduction

Type 1 diabetes (DM1) is a metabolic disorder caused by an autoimmune reaction that leads to the destruction of insulin-secreting beta cells in the pancreas. It leads to insulin deficiency and elevated levels of blood glucose (BG) referred to as hyperglycemia. Long-term complications as a consequence of chronic hyperglycemia may be microvascular and macrovascular. Retinopathy, nephropathy, and neuropathy are microvascular complications, whereas cardiovascular disease, artery inflammation and injury in the peripheral system, and cerebrovascular disease are among the macro-vascular complications^1^.

The BG of normal subjects is maintained in a narrow range of 70–180 mg/dL, which is called normoglycemia. In people with DM1, normoglycemia is achieved by the lifelong administration of exogenous insulin generally under the supervision of physicians^2^. Recent technological advancements have had a considerable effect on the management of DM1. Automatic insulin delivery (AID), also referred to as artificial pancreas (AP), systems are developed for the treatment of DM1 to overcome hypo and hyperglycemia and reduce long-term complications associated with DM1. The three core components of an AID system are a continuous glucose monitoring device (CGM) that generally provides BG measurements every 5 min, an insulin pump to continuously deliver insulin, and an algorithm to calculate the optimal insulin rate to be administered to the subject with DM1^3^.

Advancements in CGM technology make it possible to analyze glycemic trends, patterns, and key information with improved accuracy, increased duration, and mean absolute relative difference (MARD) $\le$ 10%. CGM systems can be used to calculate insulin dosing rates^4^. AID systems have been reported to be a safe and effective approach to the treatment of DM1^5^. However, optimal control of postprandial BG remains a concern for AID systems for various reasons, including significant delays in insulin action as a result of the subcutaneous route, slow response of the available insulin analogues, variability in the insulin sensitivity of DM1 subjects, and high intrapatient variability. Moreover, accurate modeling of glucose absorption is not possible because of uncertainty and intraday and interday variations. To improve glycemic performance, researchers have proposed hybrid AID (HAID) systems based on feedforward control schemes, usually proportional to the carbohydrates content (CHO) in meals^6^.

HAID systems provide automated insulin delivery via closed-loop control algorithms and patient-initiated bolus insulin delivery to compensate for announced meals based on various insulin bolus calculators^7^. HAID systems have shown improved glycemic control performance with a reduction in the risk of hypoglycemia and are among the most advanced insulin delivery systems available for DM1 subjects^8^.

The CHO content in meals is one of the main parameters and nutritional determinants of postprandial BG levels in DM1. It is recommended to accurately measure CHO for improved BG control performance^9^. However, the task of CHO counting is burdensome and prone to estimation errors, with average misestimations of around 20% in adults^10^. The quality of life in people with DM1 is negatively influenced by CHO counting and makes them less confident while interacting with peers, especially around food. To maintain the precision of the CHO count, standardized foods are more likely to be chosen by people with DM1, which can negatively affect their dietary choices^11^. Furthermore, the level of literacy required to count CHO can be an obstacle for many patients with DM1, leading to the selection of packaged processed foods over whole foods (grains, fruits, etc.) due to the relative ease provided by the nutritional information label^12^. HAID systems possess the benefit of meal announcement but they must be robust to missed meals and other factors discussed above. Therefore, a fully closed-loop AID (FAID) system is highly desirable to avoid the need for CHO counting and announcing meals in patients with DM1^13^.

Several algorithms have been proposed to automate the process of detecting meals in patients with DM1. A few of the proposals include fuzzy logic^14^, various Kalman filters^15,16^, model-based detection utilizing an autoregressive model and real-time CGM data^17^, detection of an increase in the glucose rate^18^, and artificial intelligence (AI)-based meal detection^19^. Attempts have also been made to compensate for unannounced meals. The algorithms proposed include the Kalman filter to avoid CHO counting for automatic glucose regulation^20^, disturbance observer, and feedforward compensation of unannounced meals^21^, an automatic bolus priming system^22^, and a meal absorption model for AP^23^.

Reinforcement learning (RL) is a rapidly developing field of AI that has found success in many domains. A detailed systematic review reported that advanced RL algorithms can play a vital role in developing AID systems^24^. Recently, several researchers have proposed insulin bolus calculators that exploit different models of the RL algorithm^25–27^. The reported methodologies rely on information about the CHO content in meals and the meal announcement, resulting in HAID systems.

In comparison, this work aims to develop a FAID system based on a deep reinforcement learning (DRL) insulin bolus calculator to compensate for unannounced meals and to eliminate interventions from patients with DM1. A closed-loop proportional-derivative (PD) control algorithm is used for the computation of the continuous insulin delivery rate. For the detection of meals, unscented Kalman filter (UKF) predictions are utilized based on the CGM and insulin data. The FAID system is compared to two versions of the HAID system, one utilizing the standard bolus calculator (SBC) for the compensation of meal disturbances along with CHO misestimation and the other utilizing the proposed DRL insulin bolus calculator.

## Methodology

**Figure 1:**
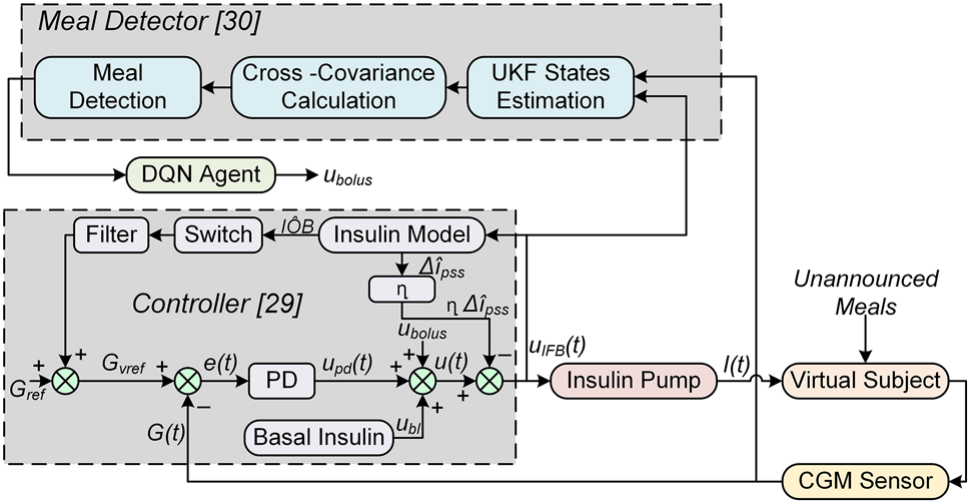
Block diagram of the proposed FAID system.

In this work, a DRL-based insulin bolus calculator is designed and integrated with a closed-loop controller and a UKF-based meal detector to compensate for unannounced meals in patients with DM1. The proposed DRL-based insulin bolus calculator is an advanced version of an algorithm published by our group^28^. The DRL algorithm is driven by meal detection and does not require information on the CHO content in meals, thereby fully closing the AID control loop. Continuous insulin delivery is achieved by a closed-loop PD controller with a safety auxiliary feedback element (SAFE) introduced in^29^. The detection of meals is based on an in-house algorithm utilizing an augmented minimal model and a UKF along with the insulin and CGM data^30^. A schematic of the overall strategy is given in Fig. 1.

### PD Controller

The control strategy involves two loops: an inner loop comprising the insulin feedback system (IFB) that relies on the PD algorithm and an outer loop that provides a safety layer to exploit the concept of insulin on board (IOB).

Three insulin components constitute the inner control action: $u_{bl}$ the basal insulin profile of the patient, $u_{bolus}$ the insulin bolus, and the PD control action resulting in insulin action given by:

1 $$\begin{aligned} u(t) = k_p \left[ e(t) + \tau _d \frac{dG(t)}{dt}\right] + u_{bl}(t) + u_{bolus} \end{aligned}$$

where $k_p~=~\dfrac{60 \times TDI}{\tau _d \times 1500}$ (U/hr) is the proportional gain, TDI is the total daily insulin, *e*(*t*) is the error in glucose concentration and $\tau _d~=~90$ (min) is the derivative time constant.

The safety layer is based on sliding mode reference conditioning (SMRC) and comprises three parts: 1) a model to estimate IOB; 2) a sliding mode referencing block (SMR); and 3) a $1^{st}$-order low-pass filter to smooth the reference adaptation. The outer safety layer modifies the reference glucose concentration ($G_{ref}$) under defined conditions to ensure that the IOB is bounded (*IOB* $\in$ $[0, {\overline{IOB}}]$). Essentially, this is accomplished by a suspension of insulin infusion caused by the controller’s reference modification. $G_{ref}$ is modified to a virtual reference $G_{vref}$ in case the estimated (${\widehat{IOB}}$) approaches dangerously or exceeds the maximum allowed IOB (${\overline{IOB}}$). This phenomenon provides robustness against delays in the subcutaneous route.

The insulin absorption model^31^ is utilized to account for the estimated IOB and is given below.

2 $$\begin{aligned} \begin{aligned} \frac{dc_1(t)}{dt} = u(t) - k_{dia}c_1(t) \\ \frac{dc_2(t)}{dt} = k_{dia}(c_1(t)-c_2(t)) \\ {\widehat{IOB}}(t) = c_1(t)+c_2(t) \end{aligned} \end{aligned}$$

where $u(t)~=~u_{pd}(t)~+~u_{bl}~+~u_{bolus}$, $c_1(t)$ and $c_2(t)$ are two compartments representing the basal and bolus IOB conditions and $k_{dia}$ is a time constant that accounts for the duration of insulin action.

The SMR block is based on the concept of invariance control^32^ with *IOB*(*t*) being the variable to be bounded and belonging to the set:

3 $$\begin{aligned} \sum = \{x(t)|s(t) = {\widehat{IOB}}(t) - {\overline{IOB}}(t) \le 0 \} \end{aligned}$$

where *x*(*t*) is the state of the system and *s*(*t*) is the sliding surface, defined as:

4 $$\begin{aligned} s(t) = {\widehat{IOB}}(t) - {\overline{IOB}}(t) + \tau \left( \dot{{\widehat{IOB}}}(t) - \dot{{\overline{IOB}}}(t)\right) \end{aligned}$$

The invariance of the region $\sum$ is achieved using the following discontinuous function.

5 $$\begin{aligned} \nu (t) = \left\{ \begin{array}{rcl} \nu ^+ &{} \text{ if } &{} s(t) > 0 \\ 0 &{} \text{ otherwise } \end{array}\right. \end{aligned}$$

Finally, the smoothness of the reference change is achieved by applying a first-order low-pass filter:

6 $$\begin{aligned} \dfrac{d\nu _f(t)}{dt} = -\lambda (\nu _f(t)-\nu (t)) \end{aligned}$$

A widely used mechanism of IFB in AP systems is also implemented. The plasma insulin concentration is estimated online; then, insulin control action is inhibited proportionally. This gives rise to a new insulin control action given by:

7 $$\begin{aligned} u_{IFB} = u(t) - \eta ({\widehat{i}}_p(t) - {\widehat{i}}_{pss}(t)) = u(t) - \eta \Delta {\widehat{i}}_{pss}(t) \end{aligned}$$

where ${\widehat{i}}_p(t)$ is the estimated value and ${\widehat{i}}_{pss}(t)$ is the steady-state estimated value of the plasma insulin concentration. $\Delta {\widehat{i}}_{pss}(t)$ is the deviation of the plasma insulin concentration from the basal infusion. Further details are presented in^29^.

### Meal Detector

The meal detector algorithm^30^ takes the rate of insulin infusion and CGM value as inputs and estimates a disturbance term via an extended minimal model utilizing the UKF. The glucose subsystem comprises Bergman equations^33^ as follows:

8 $$\begin{aligned} \dfrac{dG_{pl}(t)}{dt} = -(p_1+X(t))G_{pl}(t) + p_1G_{bl}+\dfrac{D(t)}{V_g} \end{aligned}$$

where $G_{pl}(t)$ is the blood plasma glucose concentration, *X*(*t*) reflects insulin in the remote compartment, $G_{bl}$ is basal glucose, $p_1$ is the insulin-independent rate of plasma glucose utilisation, *D*(*t*) is the disturbance term included as an extended model state, and $V_g$ is the volume distribution.

Subcutaneous glucose is represented by a first-order system^34^ as given below:

9 $$\begin{aligned} \dfrac{dG_{s}(t)}{dt} = -\dfrac{1}{\tau }G_{s}(t) + \dfrac{g}{\tau }G_{pl}(t) \end{aligned}$$

10 $$\begin{aligned} \dfrac{dX(t)}{dt} = -p_2X(t) + p_3I(t) \end{aligned}$$

where $G_{s}(t)$ is the subcutaneous glucose concentration, $\tau$ is the time constant of the system, and the static gain is represented by *g*. *X*(*t*) reflects insulin in the remote compartment, $p_2$ is the disappearance rate of remote insulin and $p_3$ captures insulin sensitivity. The insulin subsystem model is the same as that represented by equation 2, and the concentration of plasma insulin^34^ is given by:

11 $$\begin{aligned} \dfrac{dI(t)}{dt} = -k_fI(t)+\dfrac{1}{V_i}.\dfrac{S_{2}(t)}{t_{max,I}} \end{aligned}$$

where $V_i$ is the distribution volume, $k_f$ is the fractional rate of disappearance, and $t_{max,I}$ is the time to maximum absorption of insulin.

After estimation of the model states given by equations 2 and 8 to 11 through UKF, the cross-covariance is calculated between the two sequences $G_s(k)$ (from the CGM data) and $D_{diff}(k)$ (forward difference of disturbance term) over a window of specified length. $G_{s_{n}}$ and $D_{diff_{n}}$ are jointly stationary random processes, and their cross-covariance sequence is defined as the cross-correlation of mean-removed sequences^35^, as given below:

12 $$\begin{aligned} \Psi _{G_{s},D(m)} = E\{(G_s(n+m)-\mu _{G_s})(D_{diff}(n)-\mu _{D_{diff}})^*\} \end{aligned}$$

where the mean values of the random processes are represented by $\mu _{G_s}$ and $\mu _{D_{diff}}$, *E* stands for the expectation operation, and $*$ represents the complex conjugate.

Meal consumption is assumed if a predefined threshold is exceeded by the cross-covariance between $G_{s}$ and $D_{diff}$ with respect to the last three consecutive samples (15 min). As a safety measure, meals are not detected during the night period (23h–6h).

The meal detector can be tuned regarding three settings with respect to the threshold and window size for cross-covariance^30^. The three settings refer to 1) highest sensitivity (high true positives (TP)), 2) trade-off (high TP and low false positives (FP)), and 3) lowest FP. In this study, trade-off tuning is used because the highest sensitivity is prone to FP and will result in the delivery of insulin bolus at times other than meals, leading to extreme hypoglycemia. The third setting was not used because it decreases the TP substantially.

A meal detection flag is triggered if:

13 $$\begin{aligned} Meal = \left\{ \begin{array}{rl} True &{} \text{ if } c_{G_s,D_{diff}}(m) \ge T \\ {} &{}and~D_{diff}(k)> 0, \\ {} &{}and~G_s(k) - G_s(k-3) > 0, \\ False &{} \text{ otherwise. } \end{array}\right. \end{aligned}$$

where *T* is the predefined threshold and $c_{G_s,D_{dif}}(m)$ represents the raw cross-covariance, as given in^30^.

### The DRL algorithm

The problem is first formulated as a Markov decision process (MDP) to implement the training of the RL agent. An MDP is defined in terms of state space *S*, action space *A*, the transition probability $P (s_{t+1} \mid s_t, a_t)$ of the next state ($s_{t+1}$) given action ($a_t$) is taken in the current state ($s_t$), and an immediate reward $r_t$, mathematically represented as a tuple *M*(*S*, *A*, *P*, *r*). In DRL, the agent is based on a combination of RL and a category of artificial neural networks (ANNs), specifically deep neural networks (DNNs), and is termed a deep Q-network (DQN). The DQN aims to learn actions that result in the maximum total expected reward. The total expected reward can be represented as $E_R = E [r_t + \gamma r_{t+1} + \gamma ^2 r_{t+2} + . . .]$, where $\gamma \in [0, 1)$ is the discount factor defining the contribution of future rewards and $r_t$ is the immediate reward at time step *t*.

In DRL, the mapping of states into actions to be taken by the DQN is termed the policy and is represented by $\pi : S \rightarrow A$. The quality of the policy is represented by the action-value function $Q_\pi (s, a)$. The policy that leads to the maximum $E_R$ is a unique optimal policy $\pi ^*$ and results in a unique optimal action-value function $Q^*(s, a)$. In this work, a fully connected DNN is used to learn $\pi ^*$ to approximate $Q^*(s, a, \theta ) \approx Q^*(s, a)$, where $\theta$ refers to the parameters of the DNN. The final goal of training the DQN is to learn $\pi ^*$, which implies that the agent will take the best possible action in a given state. In RL, the optimal action-value function is obtained on the basis of the notion of the Bellman equation^36^ given below:

14 $$\begin{aligned} Q^*(s, a)=E_{s_{t+1}}[r+\gamma \underbrace{max}_{a}Q^*(s_{t+1}, a)\mid s,a] \end{aligned}$$

The optimal policy is obtained by dynamic programming to iteratively evaluate:

15 $$\begin{aligned} Q_{t+1}(s, a)=Q_t(s_t, a_t)+\alpha [r_t+\gamma \underbrace{max}_{a_{t+1}}Q_t(s_{t+1}, a_{t+1})\mid s,a] \end{aligned}$$

According to Bellman’s identity, $Q_t$ converges to $Q^*$ as $t \rightarrow \infty$, where $\alpha \in [0, 1)$ is the learning rate. This approach to RL (Q-Learning) requires the states to be discrete and lack generalization. Therefore, in DRL, $Q^*(s, a)$ is approximated by a nonlinear function approximator such as DNN. To estimate $Q^*(s, a)$, the DQN uses fixed Q-targets by maintaining the $Q(s, a, \theta )$ and the target ${\hat{Q}}(s, a, {\hat{\theta }})$, both having the same architecture. The two approximators improve the stability of optimization by updating the parameters of ${\hat{Q}}(s, a, {\hat{\theta }})$ periodically to the latest parameters of $Q(s, a, \theta )$^37^. The parameters are updated every 15 iterations during the training phase in the proposed algorithm.

In this work, multi-DQNs are implemented and trained. Typically, there are three meals per day, i.e., breakfast, lunch, and dinner. The protocol for meals is described later in the scenario subsection under Results. For each meal, the action space is divided into 8 subaction spaces based on the 8 ranges defined for the CGM value before meal intake. The action space is explained later in Sect. 2.3. Firstly, the DQN agents are personalized for each patient. Secondly, a DQN is trained for each subaction space, resulting in the implementation of 8 DQNs for each meal and leading to a total of 24 DQNs corresponding to three meals a day.

The motivation behind introducing a multi-DQN strategy is to obtain a personalized DRL agent for each subaction space with respect to meals. This approach will limit the learning experience of each DQN to that specific subaction space and meal, thereby providing greater chances of better performance. In summary, it is the personalization of a DQN based on the meal and the CGM value before meal intake.

**Figure 2:**
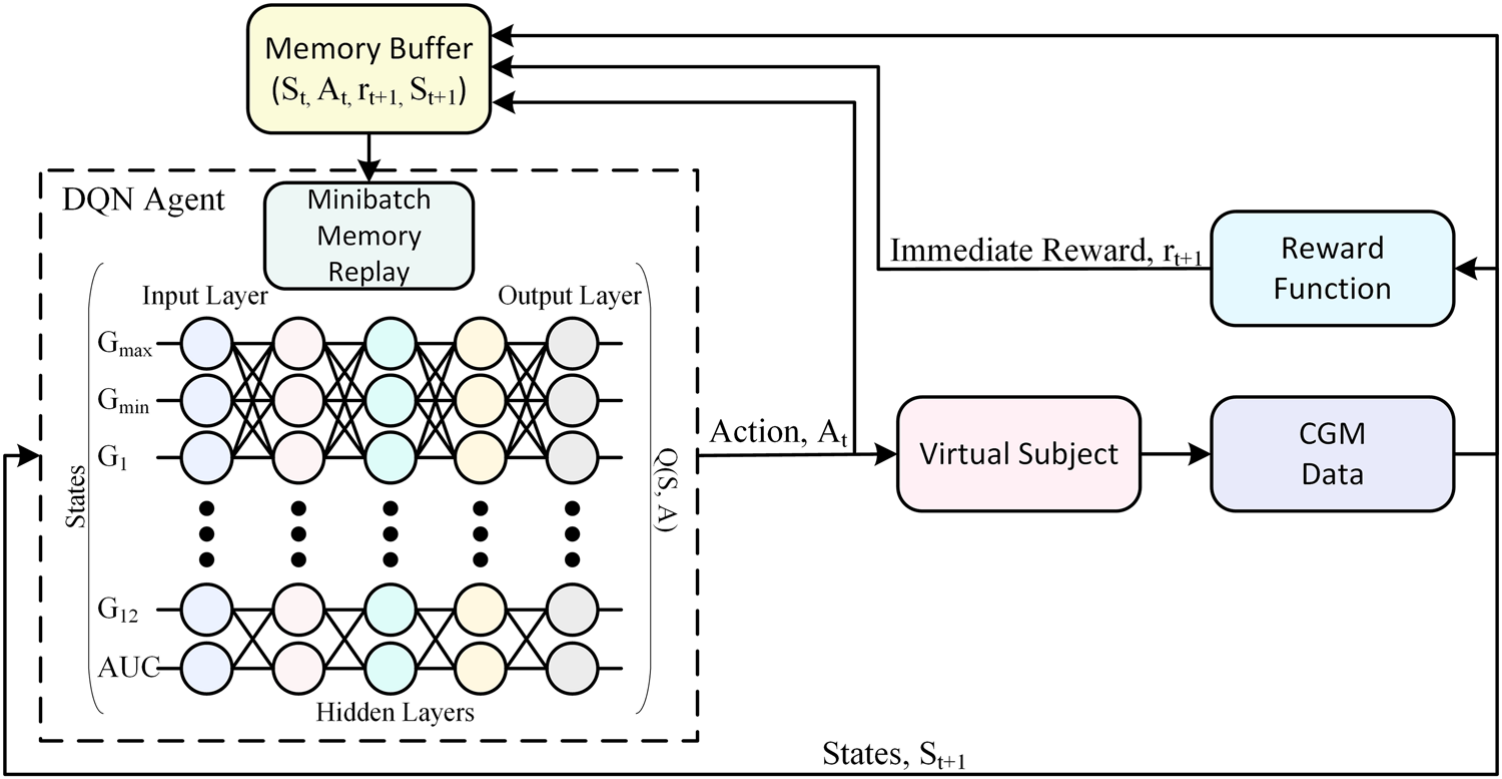
Representation of the DRL algorithm based on DQN. The states feed the DQN to approximate the optimal policy $Q^*(s, a)$. A randomly extracted mini-batch of experiences is also utilized by the DQN. The action $A_t$ corresponds to the maximum Q-value, which is the insulin bolus to be delivered to the patient. As a result, a transition occurs for the state $S_{t+1}$, and the memory buffer is updated with the new experience.

A fully connected ANN composed of three hidden layers is considered to represent a DQN for the approximation of $Q^*(s, a, \theta )$. Each hidden layer is composed of 28 nodes. The whole network consists of 5 layers, including the input and output layers. The input layer represents 15 parameters (defining the state), and the output layer shows the Q-value of each action taken in that particular state. The Q-value used in RL measures the effectiveness of the action taken in a certain state. The DQN architecture is presented in Fig. 2.

The main components of the MDP model considered in this study are explained below:

#### State space

The states are represented as the current state and the next state. DQN takes the action in the current state, which is then evaluated in the next state during the training process. In DRL, the states are continuous in nature, and discretization of states is not required. The current state is based on the pre-prandial CGM data of 4 hours. The parameters considered are the maximum CGM value, minimum CGM value, area under the curve (AUC) of the CGM data, and the 12 CGM values (1-hour data) before meal intake, summing to 15 parameters. AUC is calculated for the CGM data representing hyper or hypoglycemia only. In the next state, the same parameters are calculated based on the 4-hour postprandial CGM data, and the 12 CGM values are considered for the last hour of the postprandial window. The states are based on the CGM data, so the ANN can learn hidden patterns in the BG profile. The state space can be represented as:

16 $$\begin{aligned} S=\{G_{max}, G_{min}, G_{t_m-1}, G_{t_m-2}, G_{t_m-3},... G_{t_m-k}, AUC\} \end{aligned}$$

where $G_{max}$ is the maximum CGM value, $G_{min}$ is the minimum CGM value, $t_m$ is the meal detection time, *k* is the sample, $G_{t_m-k}$ is the CGM value at $t_m~-~k$ and *AUC* is the area under the curve over 4 hours of CGM data corresponding to hyper and hypoglycemia only.

#### Action space

**Figure 3:**
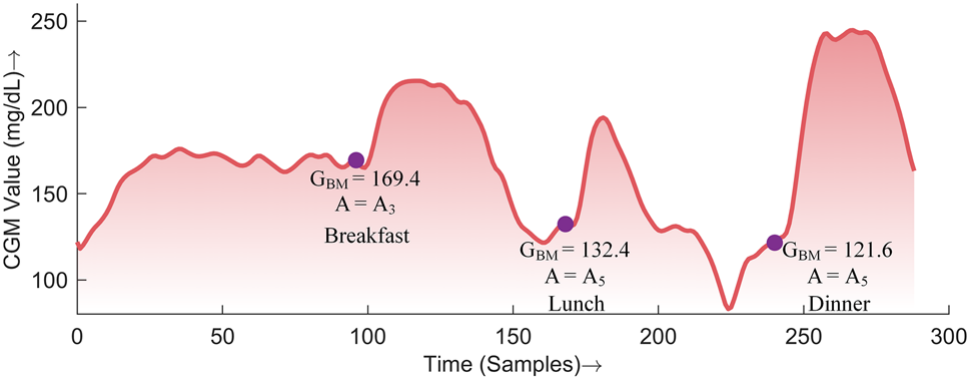
Demonstration of the selection of a subaction space based on the CGM value before a meal.

The action space for a certain meal is classified into 8 subaction spaces (SASs) corresponding to 8 different BG ranges. The number of SASs in a previous study^28^ was 7, but the number has now been increased to 8 to enhance safety based on BG before a meal and to provide greater flexibility to the agent in the choice of insulin bolus. According to the CGM value (sample) before meal intake ($G_{BM}$), belonging to one of the 8 defined ranges, the corresponding SAS is selected for action by the DQN agent. The actions considered in this study are discrete and are the bolus insulin units to be delivered to the patient, as described in^28^. The action space can be represented as:

17 $$\begin{aligned} A = \left\{ \begin{array}{ll} A_1 &{} \quad G_{BM} \ge 200 \\ A_2 &{} \quad 180 \le G_{BM}< 200 \\ A_3 &{} \quad 160 \le G_{BM}< 180 \\ A_4 &{} \quad 140 \le G_{BM}< 160 \\ A_5 &{} \quad 120 \le G_{BM}< 140 \\ A_6 &{} \quad 100 \le G_{BM}< 120 \\ A_7 &{} \quad 80 \le G_{BM}< 100 \\ A_8 &{} \quad G_{BM} < 80 \end{array} \right. \end{aligned}$$

where *A* is the action space and $A_i \mid i=1, 2... 8$ represents the SASs. $A_i$ = $\{a_1, a_2... a_j\}$, where $a_1$...$a_j$ are the bolus insulin units calculated based on the total daily insulin requirement of the patient and the value of $G_{BM}$. In this study, *j* = 15, i.e., an agent can choose among 15 actions from a chosen SAS. The selection of SAS for a single iteration is demonstrated in Fig. 3.

The insulin bolus selected as an action is further adjusted according to the bolus insulin on board (BOB) to ensure safety and avoid extreme hypoglycemic events. The adjustment can be represented as a piece-wise function:

18 $$\begin{aligned} u_{ad} = \left\{ \begin{array}{ll} a_j-{\widehat{BOB}}/k_{BOB} &{} \quad a_j>{\widehat{BOB}}/k_{BOB}~ \& ~G_{BM}\ge 180 \\ decrease~a_j~by~5\% &{} \quad a_j<{\widehat{BOB}}/k_{BOB}~ \& ~140\le G_{BM}<180 \\ decrease~a_j~by~10\% &{} \quad a_j<{\widehat{BOB}}/k_{BOB}~ \& ~120\le G_{BM}<140\\ decrease~a_j~by~20\% &{} \quad a_j<{\widehat{BOB}}/k_{BOB}~ \& ~80\le G_{BM}<120\\ a_j &{} \quad otherwise \end{array} \right. \end{aligned}$$

where $u_{ad}$ is the adjusted insulin bolus to be delivered, $a_j$ is the action chosen by the agent, ${\widehat{BOB}}$ is the estimated BOB and $k_{BOB}$ is a hyperparameter that is tuned separately for all SASs and three meals. A two-compartment model is used to estimate BOB^38^.

#### Reward function

**Figure 4:**
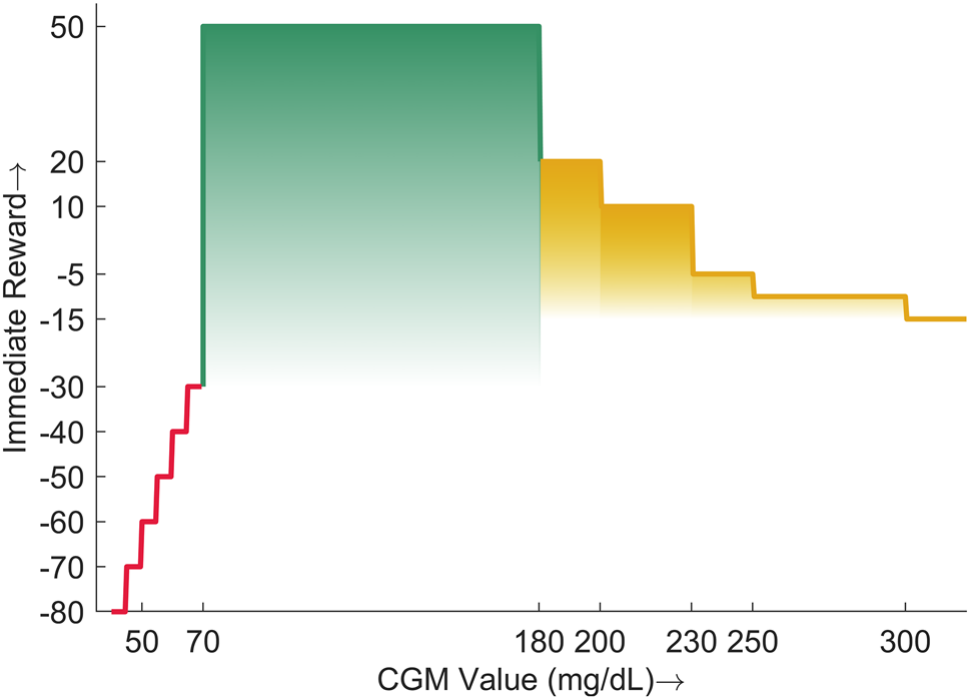
Reward function for the proposed DRL algorithm. The green region represents the immediate reward when $G_{pp}$ is in a healthy range, yellow for hyperglycemia and red for hypoglycemia.

An immediate reward is assigned to the actions of the DQN based on the next state. If the postprandial BG is in the normal range (70-180 mg/dL), a high reward is given to the DQN. If the action taken by the DQN results in hyper or hypoglycemia, the agent is penalized. The numerical values assigned to the immediate rewards are illustrated in Fig. 4 and can be expressed as a piece-wise defined function:

19 $$\begin{aligned} r_t = \left\{ \begin{array}{ll} 50 &{} \quad 70 \le G_{maxp} < 180 \\ 20 &{} \quad 180 \le G_{maxp}< 200 \\ 10 &{} \quad 200 \le G_{maxp}< 230 \\ -5 &{} \quad 230 \le G_{maxp}< 250 \\ -15 &{} \quad 250 \le G_{maxp}< 300 \\ -20 &{} \quad G_{maxp} \ge 300 \\ -30 &{} \quad 65 \le G_{minp}< 70 \\ -40 &{} \quad 60 \le G_{minp}< 65 \\ -50 &{} \quad 55 \le G_{minp}< 60 \\ -60 &{} \quad 50 \le G_{minp}< 55 \\ -70 &{} \quad 45 \le G_{minp}< 50 \\ -80 &{} \quad G_{minp} < 45 \\ \end{array} \right. \end{aligned}$$

where $G_{maxp}$ and $G_{minp}$ represent the maximum and minimum glucose values in the postprandial period, respectively. In the case of the simultaneous occurrence of $G_{maxp}$ and $G_{minp}$, the value associated with $G_{minp}$ is considered. The reward function is designed to reward the DQN agent for optimal performance, i.e., maintaining postprandial glucose in the normal range. The reward values are considered positive for mild hyperglycemia to avoid hypoglycemic episodes. There exists a trade-off between avoiding hyper and hypoglycemia, as no information on the meal content is available. On the other hand, the occurrence of hypoglycemia is penalized proportionally to the intensity of the event to avoid severe postprandial hypoglycemia.

#### Implementation

The concept of experience replay is typically used in DRL for stability and convergence of the DNN^37^. This concept is also implemented in the proposed methodology. Memory is defined for each DQN. The memory buffer (*MB*) consists of the past experiences of the agent and can be represented as:

20 $$\begin{aligned} MB=\{\xi _1, \xi _2, \xi _3,....,\xi _n\} \end{aligned}$$

where *n* is the size of the *MB* and $\xi$ is a single iteration experience given by:

21 $$\begin{aligned} \xi = \{s_t, a_t, r_t, s_{t+1}\} \end{aligned}$$

To generate the memory, a simulation is performed for 1500 days, where the actions are taken randomly and the experiences are stored in the *MB*. The size of the *MB* varies for each DQN and depends on the number of occurrences of a specific $A_i$ during the whole simulation. The *MB* is generated for each virtual patient.

A cohort of 68 virtual patients previously developed by our group is considered in this study^39^. A protocol of three meals (breakfast at 08:00 of 30-50g, lunch at 14:00 of 50–70g, and dinner at 20:00 of 60–80g) was considered during the training session. The CHO content in meals was chosen randomly from the amounts indicated. All the meals were unannounced, and the agent only took action whenever it received a positive indicator from the meal-detector. The sources of intrapatient variability included sinusoidal variations in insulin pharmacodynamics and insulin sensitivity (circadian variability) and randomness in the rate of absorption of meals^40^. An epsilon greedy policy is used to choose the action, and an immediate reward is assigned to the DQN agent according to the reward function presented in equation 19. In a single iteration, the corresponding *MB* is updated with the new experience, and the weights of the DQN are updated based on past experiences from *MB*. The loss function used to optimize the DQN’s weights is based on the Bellman equation and is given for a $k_{th}$ iteration as follows:

22 $$\begin{aligned} L_k(\theta _k) = E_{(s_t, a_t, r_t, s_{t+1})}\sim U(MB)\left[ \left( r_t+\gamma \underbrace{max}_{a_{t+1}}\left( {\hat{Q}}(s_{t+1}, a_{t+1}; \hat{\theta _k})-Q(s, a; \theta _k)\right) \right) ^2\right] \end{aligned}$$

During learning, the Q-learning updates are applied to the mini-batches $(s_t, a_t, r_t, s_{t+1})\sim U(MB)$ extracted randomly from *MB* through uniform distribution, where $\gamma$ is the discount factor, ${\hat{Q}}(s_{t+1}, a_{t+1}; \hat{\theta _k})$ is the target DQN in iteration k, whose weights $\hat{\theta _k}$ are updated periodically with the DQN $Q(s, a; \theta _k)$ weights. The DRL training algorithm implemented in this study to calculate the insulin bolus is presented in Algorithm 1. The training is performed for each patient resulting in individually trained DQN agents.

**Algorithm 1:**
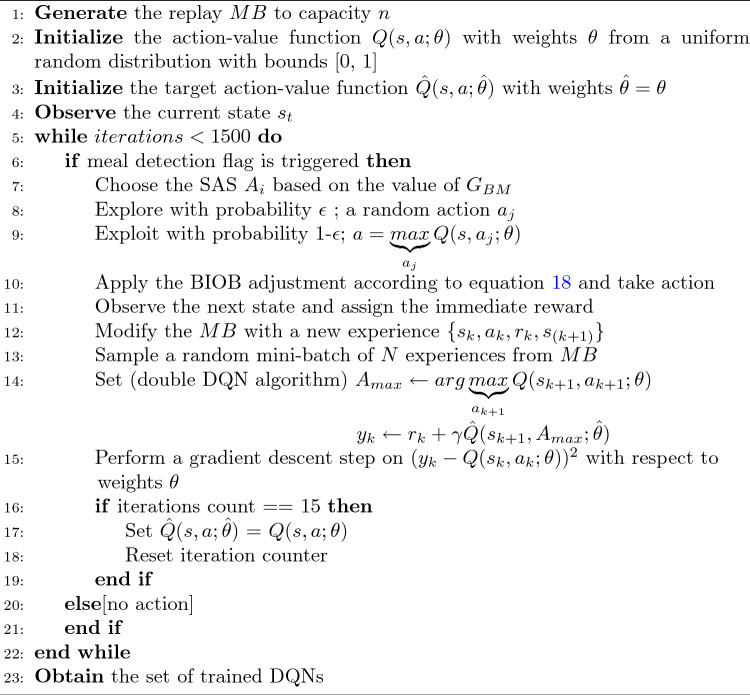
Training of Deep Reinforcement Algorithm for Insulin Bolus Calculation for the FAID

## Results

### In-silico scenario and benchmark

The virtual cohort from^39^ was used for the final testing simulations. However, the training of the DQN was not successful for one of the virtual patients. Therefore, in the subsequent analysis that patient has been removed. The simulation time for in-silico trials is 14 days. The meals delivered include breakfast at 07:00, lunch at 13:00, a snack at 17:00, and dinner at 20:00, composed of a CHO content selected randomly from 30–50g, 50–70g, 30–50, and 60–80g, respectively. During the simulations, the meal time is varied ±30 min around the time mentioned above. Variability is also incorporated, including randomness in the rate of absorption for meals, random CHO content in meals, and circadian variability in insulin sensitivity, to emulate real-life conditions^40^.

Three insulin delivery systems are compared in this study, and they all utilize a PD closed-loop controller for continuous insulin delivery. First, the HAID system is implemented utilizing SBC for the insulin bolus calculation, and the CHO misestimation error is included to be more realistic. This baseline system is represented as HAID SBC MCHO. The CHO misestimation error is incorporated as a Gaussian distribution according to the recently published methodology^41^. To implement the SBC, the parameters required are the carbohydrate-to-insulin ratio (CR) and correction factor (CF), calculated based on clinical guidelines^42^. Then, the formula for SBC used in this study is given below^43^:

23 $$\begin{aligned} u_{bolus}=\frac{CHO}{CR}+\frac{(BG_k-BG_T)}{CF}-{\widehat{IOB}} \end{aligned}$$

where $u_{bolus}$ is the bolus insulin, $BG_k$ is the CGM value at the time of delivering the bolus, $BG_T$ is the target glucose value and ${\widehat{IOB}}$ is the estimated insulin on board.

Second, the HAID system with the proposed DRL insulin bolus calculator is represented as HAID DRL. As the DRL bolus calculator is independent of the CHO content in meals, CHO misestimation is not an issue in this case. In both HAID systems, all the meals are announced, hence the name hybrid. In this case (HAID DRL), the DRL algorithm was tuned and trained in the setting of announced meals. This implies that the meal detector was not used and the insulin bolus was delivered at meal time during the training session of DQN agents. The simulation performed for generating the memory (required for the memory replay concept in the DRL algorithm) was also based on announced meals. HAID DRL is included to explicitly show the difference in the glycemic performance induced by unannounced meals.

Finally, the proposed FAID system is the main contribution of this study. The FAID system is based on the DRL algorithm for bolus insulin dosing, but all the meals are unannounced. The delivery of insulin bolus is triggered by a signal from the meal detector whenever a meal is detected.

### Comparative analysis

To draw a comparison and investigate the performance of the proposed FAID system, the outcomes of the in-silico simulations are presented in the standardized core CGM metrics, as reported in a consensus report by the American Diabetes Association (ADA) and the European Association for the Study of Diabetes (EASD)^44^.

The standardized CGM metrics and insulin information are presented in Table 1. The mean and median CGM values reported for the FAID system were statistically similar to those of the HAID systems, as indicated by the p-values. The extreme CGM values, i.e., minimum and maximum in the FAID system, were more spread, leading to a slightly higher glycemic variability, as indicated by the higher CV compared to that of the HAID systems. The FAID system achieved a similar glucose monitoring index (GMI), as reflected by the p-value.

The percentage of the CGM values (PCGM) reported for the ranges provided in Table 1 showed an overall increase of 5% in the PCGM below 70 mg/dL and above 250 mg/dL (hypoglycemia and hyperglycemia) for the FAID system. Specifically, the difference in hypoglycemia (below 70 mg/dL) was 0.9%, and that in hyperglycemia (above 250 mg/dL) was 4.1%, which is in accordance with the designed reward function. Hypoglycemia was penalized more than hyperglycemia since a hypoglycemic excursion is riskier than a hyperglycemic excursion of the same magnitude.

According to the p-values, the differences in PCGM ranges are significant, except for the tight target range (70–140 mg/dL). Importantly, all the values achieved were in the range recommended by the ADA consensus report^44^. Moreover, the glycemic risk index (GRI), a measure of the quality of glycemia based on hypoglycemia and hyperglycemia components using CGM tracings^45^, is also provided.

**Table 1:** Comparison of standardized CGM metrics and insulin data for the FAID system.

Performance Indicator	$HAID~SBC~MCHO^1$	$HAID~DRL^2$	$FAID^3$
Mean CGM (mg/dl)	153.1 (147.3 - 161.1 )	155.8 (149.7 - 160.3 )	156.1 (148 - 167.5 )
Median CGM (mg/dl)	146.7 (140.9 - 155.9 )	149.2 (144.8 - 154.9 )	147.9 (140.2 - 158.4 )
Max CGM (mg/dl)	306.7 (283.6 - 324.6 )	293.6 (265 - 347.3 )	317.7 (296.2 - 347.5 )$^{\star }$
Min CGM (mg/dl)	66.6 (43.9 - 74.7 )	72.3 (49.7 - 81.7 )	43.1 (32.3 - 61.1 )$^{\star }$
CV	25.6 (23.3 - 28.5 )	24.1 (21.9 - 28.2 )	30.6 (27.7 - 32.3 )$^{\star }$
GMI (%)	7 (6.8 - 7.2 )	7 (6.9 - 7.1 )	7 (6.8 - 7.3 )
Below 54 (%)	0 (0 - 0.3 )	0 (0 - 0.2 )	0.4 (0 - 0.9 )$^{\star }$
54 to 69 (%)	0.1 (0 - 0.5 )	0 (0 - 0.3 )	0.5 (0.2 - 1 )$^{\star }$
70 to 140 (%)	42.2 (33.7 - 48.3 )	38.9 (33.1 - 43.4 )	41.1 (33.9 - 48.1 )
70 to 180 (%)	76.2 (69.6 - 82.1 )	75.7 (71 - 81.3 )	71.2 (60.2 - 77.2 )$^{\star }$
181 to 250 (%)	19.2 (14.7 - 23.4 )	19.8 (15.5 - 24.1 )	22.6 (18.8 - 27.5 )$^{\star }$
Above 250 (%)	1.9 (1.1 - 3.1 )	1.6 (0.7 - 4 )	4.1 (1.8 - 8.4 )$^{\star }$
GRI	20.9 (16.6 - 26.5 )	20.9 (16.2 - 26.5 )	27.8 (21.6 - 41.4 )$^{\star }$
Basal Insulin (U/day)	6.3 (5 - 8 )	6.3 (5.1 - 7.8 )	8.4 (6.9 - 10 )$^{\star }$
Bolus Insulin (U/day)	22.1 (16.5 - 26.7 )	21.5 (16.7 - 26.4 )	10.1 (7.8 - 14 )$^{\star }$
TDI (U)	28.5 (23.1 - 32.3 )	27.5 (24.4 - 33.1 )	19.1 (15.5 - 23 )$^{\star }$

$^1$HAID SBC MCHO = Hybrid automatic insulin delivery (closed-loop) with standard bolus calculator and CHO misestimation.

$^2$HAID DRL = Hybrid automatic insulin delivery (closed-loop) with proposed DRL bolus calculator.

$^3$FAID = Fully automatic insulin delivery with proposed DRL bolus calculator.

$^{\star }$ p value < 0.01. The p values (FAID vs HAID SBC MCHO) are based on the Wilcoxon signed-rank test.

The performance of the FAID system is coupled with the accuracy of the meal detector and the time duration of detection. The performance metrics of the meal detector are presented in Table 2, which summarizes the populational detection performance of meals. The detection of lunch and dinner was better, as evidenced by sensitivity and true positives, whereas the snacks were barely detected. The detection of breakfast was approximately 60%. The time taken to detect a meal ranged between 30 and 40 min. As reported in Table 2 FP amounted to fewer than 1 meal in the cases of breakfast, lunch, and snacks, and none resulted in a hypoglycemic event. However, in the case of dinner, this number is approximately 2.4 meals, and a total of 8 hypoglycemic events were observed.

**Table 2:** Performance metrics of the meal detector.

Sensitivity (%)	Detection Time (min)	TP	FP	FN
Breakfast				
57.74 ± 14.43	37.92 ± 2.34	8.08 ± 2.02	0.67 ± 0.78	5.92 ± 2.02
57.14 (35.71 - 84.29)	38.75 (35 - 40)	8 (5 - 11.8)	0.5 (0 - 2)	6 (2.2 - 9)
Lunch				
95.24 ± 5.56	35 ± 0	13.33 ± 0.78	0.67 ± 0.98	0.67 ± 0.78
96.43 (85.71 - 100)	35 (35 - 35)	13.5 (12 - 14)	0 (0 - 2.9)	0.5 (0 - 2)
Snacks				
8.33 ± 5.96	29.17 ± 17.88	1.17 ± 0.83	0.33 ± 0.49	12.83 ± 0.83
7.14 (0 - 14.29)	37.5 (0 - 44.75)	1 (0 - 2)	0 (0 - 1)	13 (12 - 14)
Dinner				
95.83 ± 4.78	34.38 ± 1.55	13.42 ± 0.67	2.42 ± 1.31	0.58 ± 0.67
96.43 (86.43 - 100)	35 (30.25 - 35)	13.5 (12.1 - 14)	2 (0.1 - 4.9)	0.5 (0 - 1.9)

Values reported as mean ± standard deviation and median (25–75%).

TP, true positive; FP, false positive; FN, false negative.

**Figure 5:**
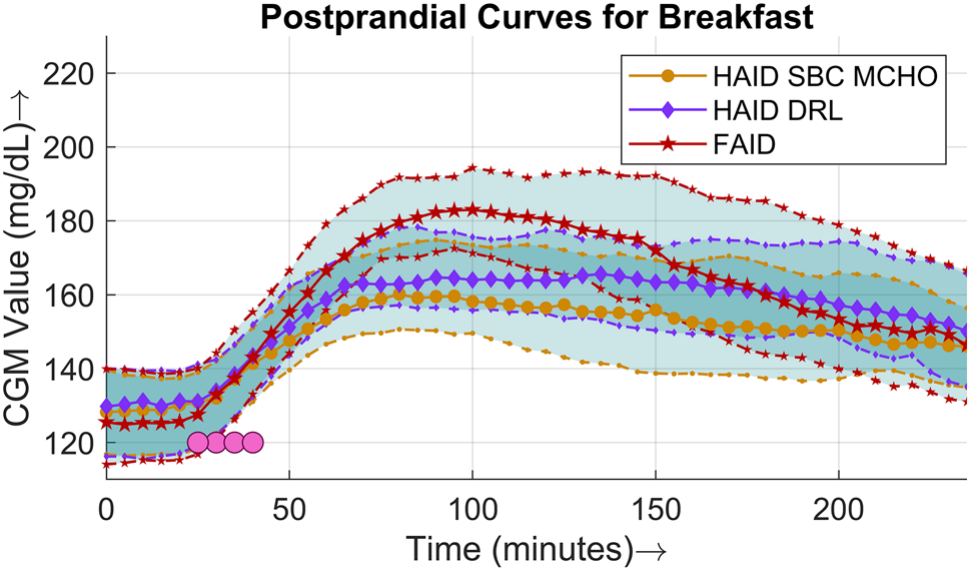
Four-hour postprandial BG curves for breakfast. The solid lines (middle curve) represent median values, whereas the dotted lines (upper and lower curves) correspond to the interquartile range of 25% and 75% respectively. The filled circles are points where meals were detected, plotted against the time of detection in minutes in the case of the FAID system.

**Figure 6:**
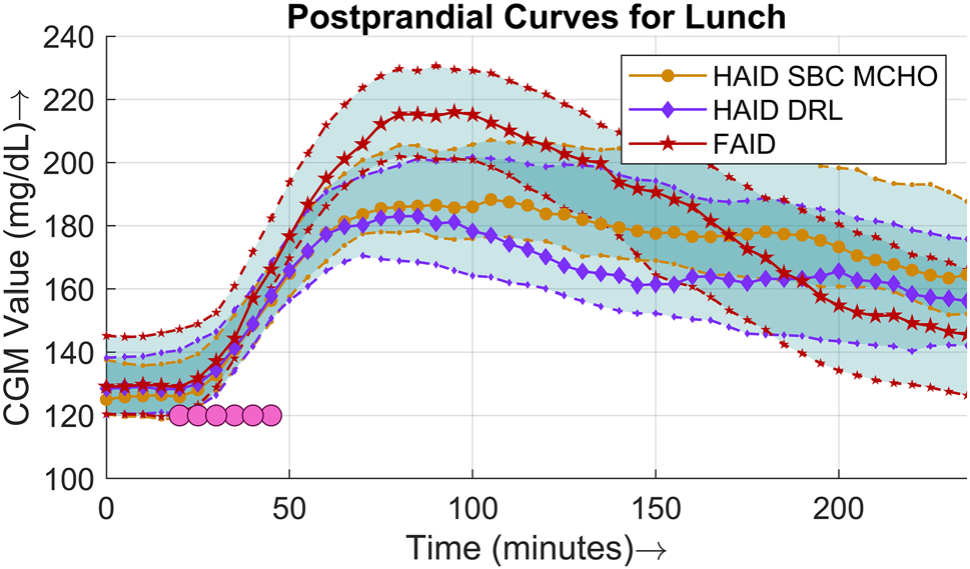
Four-hour postprandial BG curves for lunch. The solid lines (middle curve) represent median values, whereas the dotted lines (upper and lower curves) correspond to the interquartile range of 25% and 75% respectively. The filled circles are points where meals were detected, plotted against the time of detection in minutes in the case of the FAID system.

**Figure 7:**
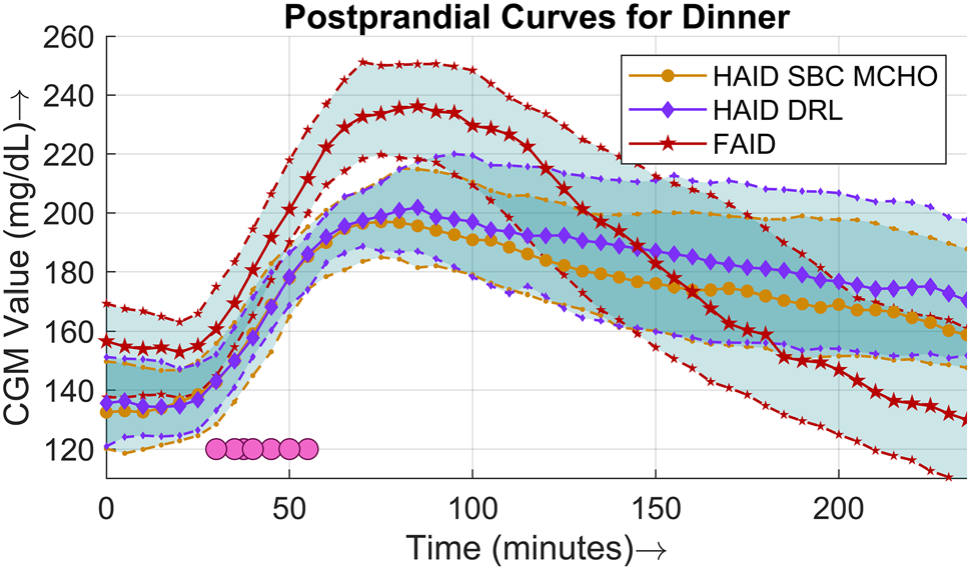
Four-hour postprandial BG curves for dinner. The solid lines (middle curve) represent median values, whereas the dotted lines (upper and lower curves) correspond to the interquartile range of 25% and 75% respectively. The filled circles are points where meals were detected, plotted against the time of detection in minutes in the case of the FAID system.

To exemplify the performance of the approach, the four-hour postprandial BG curves for each meal are illustrated in Figs. 5, 6, and 7. The BG followed a similar trajectory in all three cases. The postprandial peak BG values were higher in the case of the FAID system, reflecting the 30 to 40 min of delay in the delivery of the insulin bolus as a consequence of meal detection. The populational values of the meal detection time in minutes are represented by filled circles (pink) in the case of the FAID. Points on top of each other represent meals on different days with the same time of detection, whereas points along the x-axis represent meals with different times of detection. The time of detection is represented by the x-axis in minutes, with the meal appearing at $t~=~0$.

**Figure 8:**
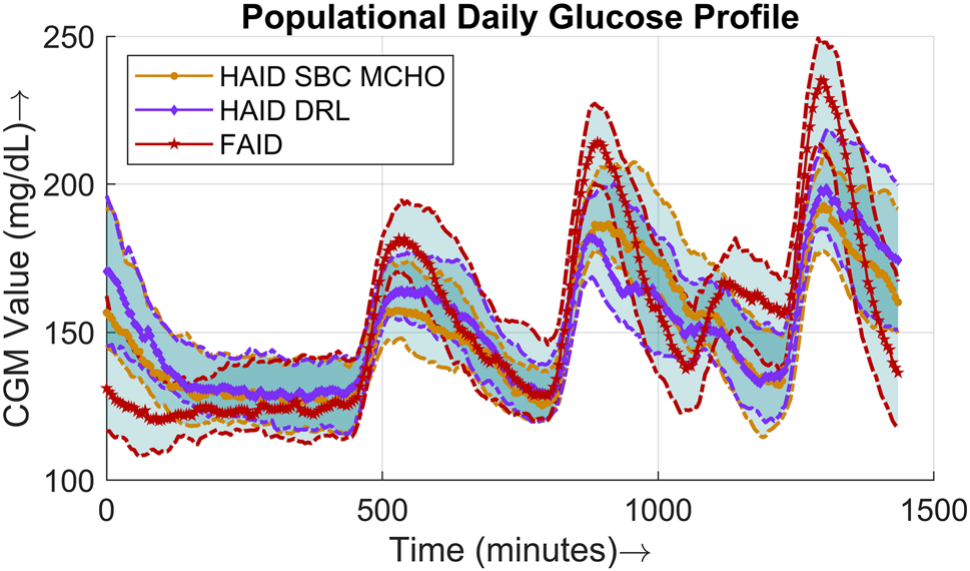
This figure shows the median daily CGM profile of the whole cohort. The day starts at 12:00 AM. The three peaks appearing are breakfast, lunch, and dinner respectively. The small spike between lunch and dinner represents the snacks. The solid lines (middle curve) represent median values, whereas the dotted lines (upper and lower curves) correspond to the interquartile range of 25% and 75% respectively.

## Discussion

The development of reliable and safe FAID systems is one of the current mainstreams in DM1 technology research. Although many disturbances affect people with DM1, such as exercise, stress or other medications, it is common practice to classify FAID systems as those that do not require meal input. To accomplish a FAID system first meal detection has to be done accurately and in a timely manner, and then compensate them. Therefore, the performance of these type of systems can be affected by two core steps: 1) detection and 2) compensation.

Several attempts have been made in the pursuit of a reliable FAID system. A learning-MPC algorithm was validated in an inpatient clinical study for a single unannounced meal in 29 patients with DM1^46^. No severe hypoglycemia was recorded, and it was suggested to extend the time of clinical trials and the number of unannounced meals in a future study. Analysis of the initial safety and efficacy of a FAID system based on a multiple-model probabilistic controller was presented for patients with DM1^47^. Thirty hours of inpatient study in 10 patients and 54 hours of supervised hotel study in 15 patients were performed, challenging the controller with unannounced meals. It was concluded that there exists a greater risk of hypoglycemia compared to that of the HAID algorithms. A meal detection and estimation module was presented, relying on the fuzzy logic algorithm^48^. The algorithm was evaluated in a retrospective study for a total of 117 meals and 11 patients. The percentage of FPs reported was 20.8%. The detector was integrated with the AP system, but the calculation of insulin bolus was also dependent on the patient’s CR. In a more recent study, an internal model control approach was used to derive a feedback controller for the FAID system and was tested in the UVa/Padova DM1 simulator. The outcome was presented in terms of the CGM curve and compared with open-loop therapy, and it was reported that the postprandial peak was reduced by approximately 8%^49^.

In this work, we have proposed a FAID system to compensate for meal disturbances by utilizing a DRL insulin bolus calculator. Three core components were integrated to implement the FAID system, i.e., a closed-loop PD controller for continuous insulin delivery, a detection algorithm for meal disturbances, and the DRL-based insulin bolus calculator. The proposed DRL insulin bolus calculator builds on top of our previous work^28^ and goes one step further. The key novelties of this paper include: 1) the complete elimination of meal announcements; 2) the improvement of the RL algorithm by using DRL based on DNNs; and 3) the integration of a closed-loop controller and meal detector algorithms together with the DRL system. Specifically, the state space and action of the DRL algorithm have been reworked and improved. One one hand, the use of DNNs allowed to describe the state space in continuous form and now it is composed of 15 continuous parameters. On the other hand, an additional subspace is also added to the action space to increase the range of actions to be chosen by the DRL agent. Additionally, on design benefits of the proposed system is that it could also accommodate announced meals without knowing the CHO content, unlike the methodologies presented in the literature. In such cases, the insulin bolus calculator could be fed by meal announcement instead of the meal detector.

### Performance analysis

The primary CGM metrics are presented in Table 1. CHO misestimation is included in the HAID with SBC to depict a real-life scenario. The absolute CGM values (mean, median, and maximum) are similar, whereas the minimum CGM is lower in the case of the FAID system because the insulin bolus calculation does not utilize CHO information and there is an inherent delay in bolus delivery due to the meal detection. The CV was slightly higher for the FAID system but was in the acceptable range of $<36\%$ as recommended by an international consensus report^44^. The GMI, an approximation of the A1C level based on the average BG from CGM^50^, was similar in all cases.

The PCGM in the tight target range ($70-140$ mg/dL) was similar, and that in the target range ($70-180$ mg/dL) was lower by 5% in the FAID system. First, the PCGM in the range below 70% accounted for approximately 1% owing to the reasons mentioned above. Second, an increase was observed in the PCGM in the range above 180 mg/dL. This increase was induced by a delay in the bolus insulin delivery proportional to the meal detection duration. Moreover, a less aggressive dosing of bolus insulin, as reflected by greater penalties for hypoglycemia, also results in a lowering of PCGM in the target range ($70-180$ mg/dL).

A comparison of the postprandial performance is explicitly presented in terms of populational postprandial BG curves for the three major meals in Figs. 5, 6, and 7. For all three meals, a similar pattern was observed, i.e., the peak was higher and the slope of the BG dip was steeper in the case of the FAID system as a consequence of the delay in insulin bolus delivery. Despite the steeper slope of the BG dip, there was no risk of severe hypoglycemic events owing to the higher peaks in the postprandial period. To show the overall daily glucose profiles Fig. 8 is presented.

**Figure 9:**
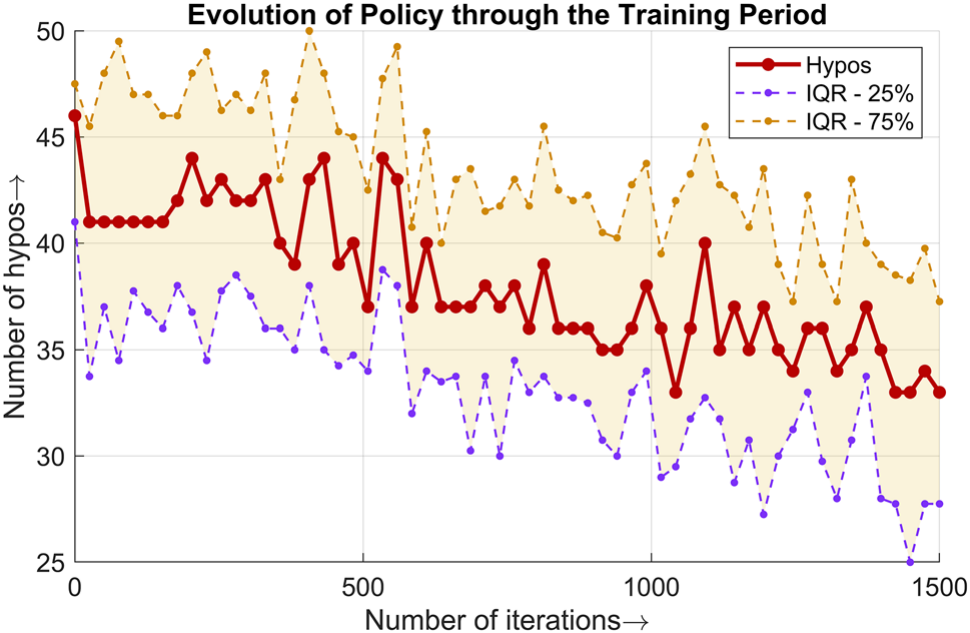
Populational number of hypoglycemia events throughout the training period lasting for 1500 iterations. An epsilon greedy policy was followed for the purpose of training.

The improvement in policy and performance of the DQN agents during the training session is presented in terms of the total number of hypoglycemia events in Fig. 9. Each point in the plot represents a median of the number of hypoglycemia events per day for all patients for 25 days. A window of 25 days was selected to highlight the trend in the number of hypoglycemia events as training progressed. During training, an epsilon greedy policy that consists of both exploitation and exploration was considered; therefore, the trend was not downward throughout, but the overall impact was. As is clear from Table 1, the time spent in hypoglycemia was approximately 1% when the trained DRL agents were deployed.

### Comparison with state of the art

Two RL algorithms are considered for comparison in this subsection. Both of the studies represent HAID systems. The RL algorithm presented in^27^ learns the programmable basal rates and the CRs for insulin bolus calculation. The simulator used for in-silio validation was based on the Hovorka model^51^. The DRL algorithm proposed in^52^ is based on double deep Q learning topology and is validated on the UVa/Padova simulator. The major advantage as compared to the algorithms presented in the literature is that our work does not require estimating the CHO content in meals and works in a fully automatic fashion. Comparison in terms of the key percentage of time ranges for CGM values is provided in the Table 3. It is evident from the table that the safety mechanisms presented in this study to avoid hypoglycemia are reflected in the results. It is not possible to make a head-to-head comparison because of the difference in the simulation environments used for the validation of the algorithms. The RL algorithms developed for other therapies such as multiple daily injections^53^ or basal insulin dosing^54^ are not considered. A comparison with the FAID systems is not provided because it is the first attempt to analyze the performance of DRL in a FAID system to the best of the author’s knowledge.

**Table 3:** Performance metrics of the meal detector.

Algorithm	Simulator	Virtual Patients	TBR	TIR	TAB
^27^	Hovorka	50	1.1	86	13
^52^	*UVa*/*Padova*	100	4.17	70.08	23.47
HAID DRL	Customized *UVa*/*Padova*	67	0	75.7	21.4
FAID	Customized *UVa*/*Padova*	67	0.9	71.2	26.7

TBR = % of CGM values below 70 mg/dL.

TIR = % of CGM values in the range 70–180 mg/dL.

TAR = % of CGM values above 180 mg/dL.

### Limitations

Although the system showed promising performance in our in-silico tests, several precautions and limitations need to be taken into account before deploying such systems. In particular, three main limitations affect this study: (1) training and testing in an in-silico environment; (2) the meal detector role on the overall performance; and (3) how to deploy the proposed system.

Firstly, we used a modified cohort of 68 patients generated based on a real cohort of people with DM1 from the Hospital Clínic de Barcelona^39^. During our training, one virtual patient was discarded due to the DRL algorithm not converging to an acceptable policy. We want to point out that, in a real life scenario not all systems work equally well or can be applicable to all different type of people with DM1. Therefore, this shows the need to perform algorithm testing and initial tuning with patient retrospective data prior to deployment.

Secondly, the performance of the FAID system was coupled with the meal detector’s accuracy and the delayed detection time. Greater accuracy and faster detection lead to better overall glycemic control performance of the FAID system. Thus, the performance metrics of the meal detector are presented in Table 2. The detection of breakfast was better but had almost $40\%$ false negatives (FNs). Lunch was very well detected and controlled as the amount of CHO in lunch was greater than that in breakfast or snacks. The snacks were rarely detected but were well compensated by the closed-loop PD controller, suggesting that no feed-forward compensation is needed for small meals. In the case of dinner, the detection was not desirable in terms of FP, which may lead to nocturnal hypoglycemia, and a total of 8 hypoglycemic events were reported. This was one of the main reasons for lower CGM values in the case of the FAID system compared to the HAID systems. Indeed there is a trade-off when adjusting the sensitivity of the meal detector to minimize FNs because it may also increase FPs. Based on two parameters of meals, the FNs of the meal detector were compensated well by the closed-loop PD controller. First, the dynamics and appearance of CHO in BG were considered, i.e., meals having slow dynamics and rate of appearance. Second, the amount of CHO in meals, i.e., meals with minimal CHO content such as snacks, was accounted for. In the above-mentioned cases, when the meals were not detected, the disturbance was partially compensated for by the closed-loop controller. Therefore, the closed-loop controller helps alleviating delay issues caused by the meal detector. The meal detector was also disabled during night periods as a safety measure. The main purpose of the meal detector was to detect and compensate for the daily meals, i.e., breakfast, lunch, and dinner. The current performance of the meal detection suggests that it will increase the overall performance in the presence of meals during night periods and will show robustness against FPs in case of no meals. The performance of the FAID system will be analyzed with the meal detector enabled all the time in future work.

Finally, deploying a DRL algorithm to real patients may pose additional risks specially due to the exploration nature of it. In this study, this is not a safety concern in the in-silico trials. However, in clinical settings, it can be dangerous, for example, the management of DM1 without taking into account safety constraints^55^. Thus, the main limitation of this study was implementation of the FAID system in a virtual environment, as clinical settings would be more challenging owing to uncertain conditions in real-life scenarios. A four-step approach suggested in^28^ can be followed to move from in-silico to clinical trials. However, a customized virtual cohort was considered. Second, the dependency of the FAID system’s performance on the meal detection algorithm limits this research. Despite having a suitable DRL insulin bolus calculator, the poor detection of unannounced meals may degrade the overall glycemic performance.

## Conclusions

In this paper, a new machine learning-based FAID system was presented by integrating a closed-loop PD controller, a UKF-based meal detector, and a DRL-driven insulin bolus calculator. The proposed DRL algorithm was based on DQN and the feature of memory replay to calculate the insulin bolus without requiring information regarding CHO content, CR, and CF, thereby paving the way for the elimination of meal announcements.

The proposed FAID system showed encouraging performance. The main objective of the FAID system is to eliminate patient intervention in the closed-loop system to avoid errors caused by CHO misestimation and to relieve the unnecessary burden on patients of calculating the CHO content.

Future research will include the use of a more sophisticated meal detector to reduce the delay induced by the meal detector as well as to minimize the effect of false positives and false negatives on the overall glycemic performance of the FAID system. Furthermore, the use of more advanced DRL algorithms will boost the performance, enabling the FAID system to compete with HAID systems.

## Data Availability

The datasets used and/or analysed during the current study are available from the corresponding author on reasonable request.
